# The *in vitro* effect of leptin on semen quality of water buffalo (*Bubalus bubalis*) bulls

**Published:** 2013

**Authors:** Amir Khaki, Rooz Ali Batavani, Gholamreza Najafi

**Affiliations:** 1 *Department of Clinical Sciences, Faculty of Veterinary Medicine, Urmia University, Urmia, Iran; *; 2 *Department of Basic Sciences, Faculty of Veterinary Medicine, Urmia University, Urmia, Iran.*

**Keywords:** Buffalo, Leptin, Semen, Sperm quality

## Abstract

The purpose of this study was to evaluate the probable effects of leptin addition in different levels to the semen extender on sperm quality (motility and motility parameters, viability, sperm membrane integrity, and DNA damage). Semen specimens were evaluated immediately after leptin addition, equilibration time and after thawing the frozen semen. Five healthy buffalo bulls (5 ejaculates from each bull) were used. Each ejaculate was diluted at 37 ˚C with tris-based extender containing 0 (control), 10, 20, 50, 100, and 200 ng mL^-1^ leptin. The diluted semen was kept 4 hr in refrigerator to reach to the equilibration time and then packed in 0.5 mL French straws and frozen in liquid nitrogen. Our results showed that, in the fresh semen, no significant difference was observed in all sperm quality parameters evaluated among all of the examined leptin concentrations. Addition of 10 ng mL^-1^ leptin into semen extender significantly preserved sperm motility, all of the motility parameters, and viability in equilibrated semen compared to that of control group. However, *in vitro *addition of 200 ng mL^-1^ leptin, significantly decreased theses parameters. In the frozen thawed semen, all leptin concentrations decreased sperm motility and viability, but significant decrease was observed in concentrations of 100 and 200 ng mL^-1^. Adding leptin to semen extender did not have any significant influence on sperm DNA damage and sperm membrane integrity in all examined groups. These findings suggest that *in vitro *addition of 10 ng mL^-1^ leptin could preserve sperm motility and viability in cooled semen of buffaloes.

## Introduction

Leptin is a 16 kDa adipokine, a pleiotropic cytokine-like hormone that primarily is secreted from adipose tissue, involved in regulation of energy homeostasis, neuroendocrine function, immunity, lipid and glucose homeostasis, fatty acid oxidation, angiogenesis, puberty and reproduction.^1^^-^^5^ Leptin plays a major role in regulation of energy balance by reducing food intake and increasing energy expenditure.^6^ Major role of leptin to control reproductive function is now firmly established.^7^^,^^8^ The ob/ob mice (lacking functional leptin-carrying homozygous mutation disrupting leptin gene) or OB-R/OB-R mice (lacking functional leptin receptor) are infertile and fail to undergo normal sexual maturation. Fertility of ob/ob mice is restored by leptin administration, indicating an effect of the hormone *per se* on reproduction.^9^^,^^10^ In leptin-deficient mice and humans which are in a permanent pre-pubertal state, leptin therapy alone is sufficient for resumption and completion of sexual development.^10^^-^^12^

In contrast to its well proven effects on female fertility, actual role of the hormone in regulatory network controlling male reproduction is not well known. Presence of leptin receptor on spermatic cells and Leydig cells suggested that leptin may play a direct regulatory role in male at level of the gonad.^13^ It was hypothesized that the net effect of leptin upon male reproductive function may depend on the circulating level of leptin.^8^ Several studies support role of leptin in regulation of gonadal functions in men^14^ indirectly via the central neuroendocrine system and directly via peripheral tissue membrane receptors.^15^^,^^16^ Some studies have indicated both positive and negative effects of leptin on gonads.^8^^,^^17^ Leptin was demonstrated to stimulate gonadotrophin releasing hormone secretion with indirect effects on the gonads via neuropeptide Y and its effect on testosterone secretion.^18^ Systemic administration of leptin or its active fragment, leptin116–130 amide, elicited FSH and LH secretion in male mice and rats, respectively.^19^^,^^20^ Central administration of leptin stimulated pituitary LH secretion in matured fasted cows.^21^ It is tempting to speculate that leptin has a negative influence on spermatogenesis due to the down-regulation of testicular testosterone production in humans, as others have demonstrated an inhibition of testosterone secretion by leptin *in vitro* in rat testis.^15^ Moreover, Von sobbe *et al*. suggested that dysfunction of testicular epithelia as found in hypergonadotrophic hypogonadism and high-grade oligozoospermia with decreased testosterone levels caused elevated semen leptin concentrations.^22^ On the other hand, it was reported that leptin may affect the secretion of inhibin B or may even be involved in the regulation of function of Sertoli cells, because there is a negative relation between leptin levels and the levels of inhibin B, which is secreted by Sertoli cells.^23^ Furthermore, Fombonne *et al*. reported that leptin inhibits of pre-pubertal Leydig cells *in vitro*.^24^

Leptin receptor was found to be present in spermatozoa more likely on acrosome, subequatorial area and either on the midpiece or on the whole tail of sperm.^25^ Regarding the fact that leptin receptor is expressed in tail of spermatozoa, a region which contributes mainly to sperm motility, the role of leptin in sperm motility is crucial.^26^ Jope *et al*. suggested that leptin might directly affect sperm via the endocrine system in the hypothalamus pituitary gonad axis, independently.^27^

For the first time Aquila *et al*. stated that Leptin was expressed in human spermatozoa.^28^ Thereafter, presence of leptin and its receptor was confirmed on boar and bull spermatozoa.^29^^,^^30^ They acclaimed that spermatozoa could secrete leptin and their discovery opened a new window in reproductive biology researches. However, some investigators do not agree this hypothesis.^31^ Reportedly, they suggested that the leptin mRNA in ejaculated spermatozoa could be remnant transcribed gene product from earlier spermatogenic stages^27^ or may be related to contamination by other cells in semen.^32^

There is little information about role of leptin in reproductive performance of farm animals, especially in buffalo bulls’ semen quality. The aim of the present study was to investigate the probable effects of *in vitro* addition of different leptin concentrations into the buffalo bulls’ semen extender on sperm motility and motility parameters [curvilinear velocity (VCL), straight line velocity (VSL), path velocity (VAP), lateral displacement (ALH), beat cross frequency (BCF), and linearity (LIN)], viability, sperm membrane integrity, and DNA damage.

## Materials and Methods

**Animals. **Twenty five semen samples were collected using a bovine artificial vagina from 5 sexually matured buffalo bulls, aged 2-4 years old, kept in the Buffalo Breeding Center, northwest of Iran, Urmia (37° 33 ´ N, 45° 4´ E). Semen sampling was achieved during the autumn (2011) and winter (2012). First mount ejaculated semen was collected weekly intervals, at 9-11 am. However, in the case of poor semen quality, the second mount ejaculate was taken. The semen samples which had 4-5 gross motility score at 100× magnification (0 = cell present without motility; 5 = very rapid dark swirls) were chosen for experiment.

**Semen preparation. **Each ejaculate was divided into 6 portions and diluted at 37 ˚C with tris-based extender (tris 2.660 g, glucose 1.200 g, citric acid 1.390 g, cysteine 0.139 g, doubled distilled water up to 100 mL) containing 0 (control), 10, 20, 50, 100 and 200 ng mL^-1^ leptin (Sigma Aldrich Co., St. Louis, MO, USA). Then, semen samples (6 portions per ejaculate) were stored in 4 ˚C for 4 hr on equilibration chamber. Finally, the semen was packed in 0.5 mL French straws and frozen using method of Rasul *et al*. in liquid nitrogen and stored till analysis.^33^

**Sperm quality assessment. **The sperm motility and viability were assessed in equilibrated semen and frozen-thawed semen in 37 ˚C water bath for 40 sec.^34^ Sperm motility and its parameters was estimated using a computer assisted sperm analysis (CASA) (Version 6, HFT CASA, Hoshmand Fannavar, Amirkabir Medical Engineering Co., Tehran, Iran) with a warmed microscope stage at 37 ˚C on a pre-warmed slide. A total number of 200 spermatozoa were analyzed in several microscopic fields in each specimen. Viability of spermatozoa was estimated using eosin-nigrosin staining method^35^ by observing 200 spermatozoa in light microscopic fields (Model BX41, Olympus, Tokyo, Japan).

**Fig. 1:**
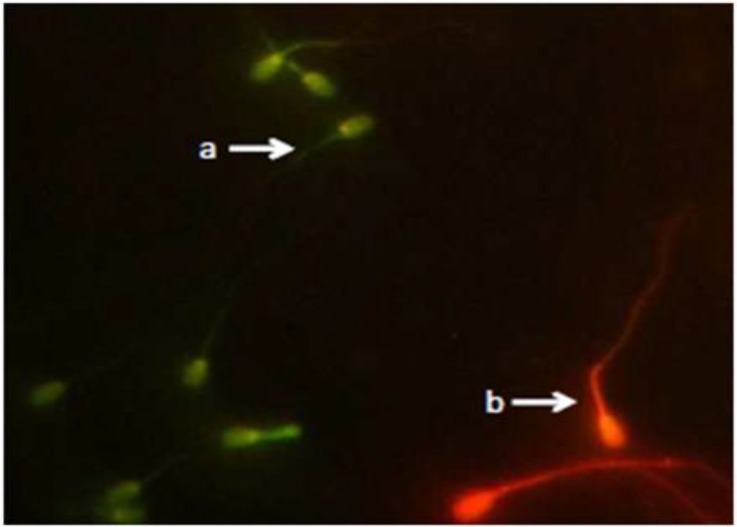
Acridine orange staining for detecting DNA damage of spermatozoa; **a)** spermatozoid with normal DNA, **b)** spermatozoid with damaged DNA

Based on Katayose *et al*. DNA damage was detected employing Acridine orange staining technique.^36^ In brief, medium-thick smears of sperm on the glass slides were air dried, fixed for 2 hr in freshly prepared Carnoy’s solution (methanol ⁄ glacial acetic acid), air dried again, and stained with acidic work solution containing 19% Acridine orange [3, 6-bis (dimethylamino) acridine, hemi (zinc chloride) salt, Sigma Aldrich Chemical Co., St. Louis, MO, USA]. All slides were examined on a fluorescence microscope (Model GS7, Nikon Co., Tokyo, Japan). A total of 200 cells were counted on each slide and classified by type as green or red based on differences in their fluorescent color (Fig. 1).

Sperm membrane integrity assessed by the hypo-osmotic swelling test (HOST), as described by Jeyendran *et al*.^37^ In brief, the hypo-osmotic solution with osmotic pressure = 150 mOsmol kg^-1^ (Osmomat 030; Gonotec, Berlin, Germany) was prepared by dissolving 0.73 g sodium citrate and1.35 g fructose in 100 ml of distilled water. Hypo-osmotic solution (500 µL) was mixed with 50 µL of semen and incubated at 37 ˚C for 40 min. After incubation, a drop of semen sample was examined and 200 spermatozoa were counted in at least 5 different fields for their swelling characterized by coiled tail indicating intact plasma membrane.

## Results

**Equilibrated semen. **Addition of 10 ng mL^-1^ leptin to semen extender preserved significantly sperm motility, VCL, VSL, VAP, ALH, BCF, LIN, and viability compared with control group and other leptin concentrations in diluted equilibrated semen (*p* ≤ 0.05). Conversely, addition of 200 ng mL^-1^ leptin to semen extender decreased significantly sperm motility, VCL, VSL, VAP, ALH, BCF, LIN, and viability compared with control group (*p* ≤ 0.05). The number of fragmented DNA cells in all groups was not statistically different from control group (Tables 1 and 2).

**Frozen-thawed semen. **The highest sperm motility (40.7 ± 1.8%) and viability (60.8 ± 1.4%) were observed in frozen-thawed semen in control group. Indeed, addition of leptin to semen extender decreased sperm motility and viability in all experimental groups and significant decrease were observed with addition of 100 and 200 ng mL^-1^ leptin compared with those of the base extender (*p *< 0.05). There was no statistically significant difference among all groups in the number of fragmented DNA sperm cells in frozen thawed semen (Tables 1 and 2).

**Table 1:** Effect of different leptin concentrations on sperm motility parameters (Mean ± SEM) in fresh, equilibrated and frozen thawed semen

	**Leptin concentration ** **(ng mL** ^-1^ **)**	**Progressive motility (%)**	**VCL ** **(µm sec** ^−1^ **)**	**VSL ** **(µm sec** ^−1^ **)**	**VAP ** **(µm sec** ^−1^ **)**	**ALH ** **(µm)**	**BCF ** **(Hz)**	**LIN ** **(%)**
**Fresh**								
	0	83.80 ± 0.90 a	54.20 ± 1.00 a	30.20 ± 0.60 a	34.60 ± 0.60 a	2.0 0 ± 0.04 a	6.30 ± 0.10 a	50.70 ± 0.70 a
	10	84.10 ± 0.80 a	54.50 ± 0.90 a	30.60 ± 0.60 a	34.80 ± 0.60 a	2.00 ± 0.03 a	6.20 ± 0.10 a	50.80 ± 0.80^ a^
	20	83.30 ± 1.00 a	53.30 ± 1.20 a	30.20 ± 0.70 a	34.20 ± 0.80 a	2.00 ± 0.05 a	6.10 ± 0.10 a	50.80 ± 0.90 a
	50	83.20 ± 1.10 a	53.10 ± 1.30 a	30.30 ± 0.70 a	34.20 ± 0.80 a	2.00 ± 0.05 a	6.20 ± 0.10 a	51.40 ± 0.80 a
	100	83.20 ± 1.10 a	53.30 ± 1.20 a	30.10 ± 0.80 a	34.10 ± 0.80 a	2.00 ± 0.04 a	6.10 ± 0.10^ a^	50.40 ± 1.00 a
	200	82.50 ± 1.50 a	52.00 ± 1.70 a	30.20 ± 0.90 a	33.80 ± 1.00 a	1.90 ± 0.06 a	6.20 ± 0.10 a	51.60 ± 1.00^ a^
**Equilibrated**								
	0	69.00 ± 2.00 b	38.60 ± 2.20 b	22.30 ± 1.00 b	25.30 ± 1.30 b	1.50 ± 0.07 b	5.20 ± 0.10 b	42.10 ± 1.30 b
	10	77.30 ± 2.10 a	46.90 ± 2.00 a	27.00 ± 1.20 a	30.00 ± 1.40 a	1.70 ± 0.07 a	5.80 ± 0.10 a	46.40 ± 1.60 a
	20	68.40 ± 2.10 b	38.40 ± 1.90 b	22.10 ± 1.10 b	24.60 ± 1.30 bc	1.40 ± 0.06 b	5.10 ± 0.10 b	41.80 ± 1.40 b
	50	66.10 ± 1.80 b	36.00 ± 1.80 b	21.00 ± 0.90 b	23.40 ± 1.00 bc	1.40 ± 0.06 b	5.00 ± 0.10 b	39.90 ± 1.20 b
	100	63.20 ± 1.90 b	34.50 ± 1.80 b	19.40 ± 1.00 b	21.50 ± 1.10 c	1.30 ± 0.05 b	4.90 ± 0.10 b	37.90 ± 1.30 bc
	200	56.00 ± 1.30 c	27.60 ± 1.90 c	15.70 ± 0.70 c	17.70 ± 0.70 d	1.10 ± 0.03 c	4.40 ± 0.10 c	35.50 ± 1.30 c
**Frozen thawed**								
	0	40.70 ± 1.80 a	18.80 ± 1.00 a	10.30 ± 0.60 a	11.90 ± 0.60 a	0.70 ± 0.05 a	3.00 ± 0.10 a	24.40 ± 1.10 a
	10	40.30 ± 1.50 a	19.30 ± 1.00 a	10.50 ± 0.50 a	12.10 ± 0.60 a	0.70 ± 0.04 a	3.00 ± 0.10 a	24.00 ± 0.09 a
	20	39.20 ± 1.70 a	17.60 ± 0.90 a	9.60 ± 0.60 a	11.20 ± 0.60 a	0.70 ± 0.04 a	2.80 ± 0.10 a	23.70 ± 1.00 a
	50	37.20 ± 1.20 ab	17.20 ± 0.70 a	9.40 ± 0.50 ab	10.90 ± 0.50 ab	0.70 ± 0.03 ab	2.70 ± 0.10 ab	22.20 ± 0.70 ab
	100	32.60 ± 2.10 bc	13.60 ± 0.90 b	7.80 ± 0.70 bc	9.30 ± 0.70 bc	0.50 ± 0.05 bc	2.20 ± 0.20 bc	19.70 ± 1.20 bc
	200	28.30 ± 1.90 c	12.20 ± 0.90 b	6.40 ± 0.60 c	7.70 ± 0.60 c	0.40 ± 0.04 c	1.80 ± 0.10 c	17.20 ± 1.20 c

a,b,c Superscript letters represents a significant difference (*p* < 0.05) within columns.

**Table 2:** Effect of different leptin concentrations on sperm viability, DNA damage, and membrane integrity (Mean ± SEM) in fresh, equilibrated and frozen thawed semen

	**Leptin concentration (ng mL** ^-1^ **)**	**Viability** **(%)**	**DNA damage** **(%)**	**HOST** **(%)**
**Fresh**				
	0	87.00 ± 0.70 a	1.80 ± 0.10 a	88.80 ± 0.90 a
	10	87.80 ± 0.70 a	1.70 ± 0.10 a	88.10 ± 1.80 a
	20	86.90 ± 0.90 a	1.80 ± 0.10 a	86.10 ± 1.20 a
	50	86.80 ± 0.60 a	1.80 ± 0.10 a	87.50 ± 1.50 a
	100	86.40 ± 0.80 a	1.80 ± 0.10 a	86.20 ± 1.10 a
	200	85.50 ± 0.80 a	1.90 ± 0.10 a	85.20 ± 1.00 a
**Equilibrated**				
	0	77.90 ± 1.50 b	3.30 ± 0.20 ab	77.50 ± 1.30 a
	10	85.40 ± 1.30 a	3.00 ± 0.20 b	81.20 ± 1.00 a
	20	76.10 ± 1.60 b	3.80 ± 0.30 ab	80.50 ± 1.30 a
	50	74.60 ± 1.60 b	3.00 ± 0.20 b	79.10 ± 1.50 a
	100	73.50 ± 1.60 bc	4.10 ± 0.30 a	79.50 ± 1.20 a
	200	69.50 ± 1.70 c	4.10 ± 0.20 a	76.00 ± 1.50 a
**Frozen thawed**				
	0	60.80 ± 1.40 a	11.00 ± 0.30 a	58.40 ± 1.50 a
	10	60.50 ± 1.80 a	10.50 ± 0.40 a	60.50 ± 1.00 a
	20	58.70 ± 1.40 a	10.90 ± 0.30 a	60.20 ± 1.60 a
	50	57.30 ± 1.60 ab	11.20 ± 0.30 a	57.30 ± 1.20 a
	100	52.50 ± 1.90 bc	11.70 ± 0.20 a	58.50 ± 1.10 a
	200	50.40 ± 1.80 c	11.90 ± 0.30 a	56.20 ± 1.30 a

a,b,c Superscript letters represents a significant difference (*p* < 0.05) within columns in each tables.

## Discussion

One of the most important goals in any buffalo farm is achieving high reproductive rates to accelerate genetic improvement, which could be performed using artificial reproduction tools. During the past decade remarkable achievements have been made in the field of buffalo assisted reproduction. In commercial livestock breeding, artificial insemination plays a major role in terms of health and economic production. Therefore, the most important challenge is availability of high quality semen from a progeny tested bull.

Although the normal values of leptin have positive effects on male reproductive activity, excess levels of leptin resulting from increased secretion from adipose tissue are known to have a deleterious effect on sperm production and secretion of androgens by Leydig cells.^38^^,^^39^ Given that the levels of leptin in both serum and seminal plasma are significantly raised in non-obstructive azoospermic, astheno-zoospermic and oligoasthenozoospermic patients than those of normozoospermic fertile patients, it has been proposed that the increased serum leptin might directly affect testicular function to reverse spermatogenic dysfunction.^14^^,^^22^^,^^31^^,^^40^

It has been reported that there is a negative correlation in leptin levels among seminal plasma, sperm concentration and motility parameters.^31^^,^^41^^-^^43^ However, some investigators found no correlation between seminal plasma leptin and physical characteristics of semen samples.^26^^,^^38^^,^^40^^,^^44^ But, Lampiao and du Plessis suggested that *in vitro* leptin significantly increased total motility, progressive motility and acrosome reaction as well as nitric oxide production in human spermatozoa.^45^

Since there are controversial reports about effect of leptin on semen parameters in human and some laboratory animals, the present study was conducted to add different concentrations of the hormone into the buffalo semen extender to evaluate *in vitro* effect of leptin on water buffaloes sperm motility, viability, and DNA damage in equilibrated and frozen thawed semen. Our findings showed that addition of 10 ng mL^-1^ leptin into semen extender in equilibrated semen preserved sperm motility, motility parameters such as VCL VSL, VAP, ALH, BCF, LIN, and viability compared to those of the control group. This result is in agreement with the study of Lampiao and du Plessis which showed that *in vitro *leptin had beneficial effects on human sperm function and concluded that the hormone could play a role in enhancing the fertilization capacity of human spermatozoa via increasing motility and acrosome reaction.^45^ Moreover, jorsaraei *et al*. reported that administration of 30 ng mL^-1^ leptin has a positive but not significant effect on human sperm motility after 4 hr incubation.^46^

Our findings showed that leptin decreased quality of frozen thawed semen. Li *et al*. found no significant difference in all the CASA motility parameters determined and percentages of capacitated and acrosome-reacted spermatozoa in seminal plasma concentration of leptin in human ejaculated semen.^26^ However, Aquila *et al*. reported that when washed pooled human sperm from normal samples were treated with leptin and incubated under uncapacitating condition, both cholesterol efflux and protein tyrosine phosphorylation were increased and they hypo-thesized an action of leptin in modulating sperm energetic substrate availability during capacitation.^28^ In addition, Aquila *et al*. also showed similar findings in leptin-treated pig spermatozoa.^47^ Additionally, Aquila *et al*. demonstrated that sperm could secret leptin and suggested that sperm had ability to modulate its metabolism based on its energy needs independent of systemic leptin expression.^28^ This may represent a protective mechanism in male reproduction to guarantee the accumulation of energy substrates to maintain the gamete fertilizing capability.^28^^,^^48^

Regarding these findings we can deduce when we add leptin *in vitro *to the semen extender, capacitation process may be occurred in spermatozoa in equilibration or even earlier; and when the semen is frozen thawed, performance of the spermatozoa may be reduced.

 Since leptin has no effect on sperm DNA damage in both equilibrated and frozen thawed semen, we could suggest that there is no correlation between *in vitro *addition of leptin in semen extender and fragmented DNA in buffalo bull sperm. 

In conclusion, the results of the present study showed that *in vitro* addition of 10 ng mL^-1^ leptin to semen extender preserved sperm motility and viability in equilibrated semen compared to those of the control group which can be helpful for insemination of cooled fresh semen collected from buffalo bulls suffering from decreased sperm motility. However, all concentrations of leptin decreased these parameters in frozen thawed semen which could be due to the effect of leptin on capacitation and hyper-activation of sperm in equilibrated semen which consequently decreased spermatozoa viability and motility in frozen thawed semen in water buffalo bulls. We found no correlation between *in vitro* leptin and buffalo bulls’ sperm fragmented DNA. Beside the *in vitro* effect of leptin on semen quality, it seems that leptin concentration in seminal plasma and blood serum of buffalo bull could have influence on the semen quality, which needs further investigations. 

## References

[1] Du Plessis SS, Cabler S, McAlister DA (2010). The effect of obesity on sperm disorders and male infertility. Nat Rev Urol.

[2] Mammi C, Calanchini M, Antelmi A (2012). Androgens and adipose tissue in males: A complex and reciprocal interplay. Int J Endocrinol.

[3] Dallongeville J, Fruchart JC, Auwerx J (1998). Leptin, a pleiotropic hormone: physiology, pharmacology, and strategies for discovery of leptin modulators. J Med Chem.

[4] Cunningham MJ, Clifton DK, Steiner RA (1999). Leptin’s actions on the reproductive axis: perspectives and mechanisms. Biol Reprod.

[5] Quinton ND, Smith RF, Clayton PE (1999). Leptin binding activity changes with age: The link between leptin and puberty. J Clin Endocrinol Metab.

[6] Bray GA, York DA (1997). Leptin and clinical medicine: a new piece in the puzzle of obesity. J Clin Endocrinol Metab.

[7] Rosenbaum M, Liebel RL (1998). Leptin: A molecule integrating somatic energy stores, energy expenditure and fertility. Trends Endocrinol Metab.

[8] Caprio M, Fabbrini E, Isidori AM (2001). Leptin in repro-duction. Trends Endocrinol Metab.

[9] Hileman SM, Pierroz DD, Masuzaki H (2002). Characterization of short isoforms of the leptin receptor in rat cerebral microvessels and of brain uptake of leptin in mouse models of obesity. Endocrinol.

[10] Mounzih K, Lu R, Chehab FF (1997). Leptin treatment rescues the sterility of genetically obese ob/ob males. Endocrinol.

[11] Chehab FF, Lim ME, Lu R (1996). Correction of the sterility defect in homozygous obese female mice by treatment with the human recombinant leptin. Nat Genet.

[12] Farooqi IS, Jebb SA, Langmack G (1999). Effects of recombinant leptin therapy in a child with congenital leptin deficiency. N Engl J Med.

[13] Hoggard N, Mercer JG, Rayner DV (1997). Localization of leptin receptor mRNA splice variants in murine peripheral tissues by RT-PCR and in situ hybridization. Biochem Biophys Res Commun.

[14] Steiman N, Gamzu R, Yogev L (2001). Serum leptin concentrations are higher in azoospermic than in normozoospermic men. Fertil Steril.

[15] Tena-Sempere M, Pinilla L, Gonz´alez LC (1999). Leptin inhibits testosterone secretion from adult rat testis in vitro. J Endocrinol.

[16] Caprio M, Isidori AM, Carta AR (1999). Expression of functional leptin receptors in rodent Leydig cells. Endocrinol.

[17] Clarke IJ, Henry BA (1999). Leptin and reproduction. Rev Reprod.

[18] Glander HJ, Lammert A, Paasch U (2002). Lepin exits in tubuli seminiferi and in seminal plasma. Andrologia.

[19] Barash IA, Cheung CC, Weigle DS (1996). Leptin is a metabolic signal to the reproductive system. Endocrinol.

[20] Gonzalez LC, Pinilla L, Tena-Sempere M (1999). Leptin 116-130 stimulates prolactin and LH secretion in fasted adult male rats. Neuro Endocrinol.

[21] Amstalden M, Garcia MR, Stanko RL (2002). Central infusion of recombinant ovine leptin normalizes plasma insulin and stimulates a novel hypersecretion of luteinizing hormone after short-term fasting in mature beef cows. Biol Reprod.

[22] Von Sobbe HU, Koebnick C, Jenne L (2003). Leptin concentrations in semen are correlated with serum leptin and elevated in hypergonadotrophic hypo-gonadism. Andrologia.

[23] Banks WA, McLay RN, Kastin AJ (1999). Passage of leptin across the blood-testis barrier. Am J Physiol.

[24] Fombonne J, Charrier C, Goddard I (2007). Leptin-mediated decrease of cyclin A2 and increase of cyclin D1 expression: relevance for the control of prepubertal rat Leydig cell division and differentiation. Endocrinol.

[25] De Ambrogi M, Spinaci M, Galeati G (2007). Leptin receptor in boar spermatozoa. Int J Androl.

[26] Li HW, Chiu PC, Cheung MP (2008). Effect of leptin on motility, capacitation and acrosome reaction of human spermatozoa. Int J Androl.

[27] Jope T, Lammert A, Kratzsch J (2003). Leptin and leptin receptor in human seminal plasma and in human spermatozoa. Int J Androl.

[28] Aquila S, Gentile M, Middea E (2005). Leptin secretion by human ejaculated spermatozoa. J Clin Endocrinol Metab.

[29] Aquila S, Rago V, Guido C (2008). Leptin and leptin receptor in pig spermatozoa: Evidence of their involvement in sperm capacitation and survival. Reprod.

[30] Abavisani A, Baghbanzadeh A, Shayan P (2011). Leptin mRNA in bovine spermatozoa. Res Vet Sci.

[31] Ishikawa T, Fujioka H, Ishimura T (2007). Expression of leptin and leptin receptor in the testis of fertile and infertile patients. Andrologia.

[32] Hatami-Baroogh L, Razavi S, Zarkesh-Esfahani H (2010). Evaluation of the leptin receptor in human spermatozoa. Reprod Biol Endocrinol.

[33] Rasul Z, Anzar M, Jalali S (2000). Effect of buffering system on post-thaw motion characteristics, plasma membrane integrity and acrosome morphology of buffalo spermatozoa. Anim Reprod Sci.

[34] Nebel RL, Youngquist RS, Threlfall WR (2007). Techniques for artificial insemination of cattle with frozen-thawed semen. Current therapy in large animal theriogenology.

[35] Barth AD, Youngquist RS, Threlfall WR (2007). Evaluation of potential breeding soundness of the Bull. Current therapy in large animal theriogenology.

[36] Katayose H, Yanagida K, Hashimoto S (2003). Use of diamide-acridine orange fluorecence staining to detect aberrant protamination of human-ejaculated sperm nuclei. Fertil Steril.

[37] Jeyendran RS, Van der ven HH, Perez Pelaez M (1984). Development of an assay to assess the functional integrity of the human sperm membrane and its relationship to other semen characteristics. J Reprod Fertil.

[38] Tena-Sempere M, Barreiro ML (2002). Leptin in male reproduction: the testis paradigm. Mol Cell Endocrinol.

[39] Isidori AM, Caprio M, Strollo F (1999). Leptin and androgens in male obesity: evidence for leptin contribution to reduced androgen levels. J Clin Endocrinol Metab.

[40] Zorn B, Osredkar J, Meden-Vrtovec H (2007). Leptin levels in infertile male patients are correlated with inhibin B, testosterone and SHBG but not with sperm characteristics. Int J Androl.

[41] Hanafy S, Halawa FA, Mostafa T (2007). Serum leptin correlates in infertile oligozoospermic males. Andrologia.

[42] Jorsaraei SGA, Shibahara H, Yustawati A (2010). The Leptin concentrations in seminal plasma of men and its relationship to semen parameters. Iran J Reprod Med.

[43] Chen B, Guo JH, Lu YN (2008). Leptin and varicocele-related spermatogenesis dysfunction: animal experiment and clinical study. Int J Androl.

[44] Camina JP, Lage M, Menendez C (2002). Evidence of free leptin in human seminal plasma. Endocrine.

[45] Lampiao F, du Plessis SS (2008). Insulin and leptin enhance human sperm motility, acrosome reaction and nitric oxide production. Asian J Androl.

[46] Jorsaraei SGA, Shibahara H, Ayustawati A (2008). The in-vitro effects of nicotine, cotinine and leptin on sperm parameters analyzed by CASA system. Iran J Reprod Med.

[47] Aquila S, Giordano F, Guido C (2011). Nitric oxide involvement in the acrosome reaction triggered by leptin in pig sperm. Reprod Biol Endocrinol.

[48] Wabitsch M, Ballauff A, Holl R (2001). Serum leptin, gonadotropin, and testosterone concentrations in male patients with anorexia nervosa weight gain. J Clin Endocrinol Metab.

